# Ether, Chloroform or Nitrous Oxide

**Published:** 1890-04

**Authors:** George R. Fowler

**Affiliations:** Surgeon to M. E. Hospital and to St. Mary's Hospital, Brooklyn.


AMERICAN JOURNAL

—OF—

DENTAL SCIENCE.

VOL. XXIII. BALTIMORE, APRIL, 1890. NO. 12.

ARTICLE I.

### ETHER, CHLOROFORM OR NITROUS OXIDE.

BY GEORGE R. FOWLER, M. D.,

[Surgeon to M. E. Hospital and to St. Mary's Hospital, Brooklyn.]

Apropos of the recent discussion before the Brooklyn Surgical Society, it may be said that the subject of anæsthetics ever since their discovery and first introduction, now several decades ago, has been a most attractive and absorbing theme to thoughtful surgeons. From time to time new experience and new conclusions are announced, and a change of front as regards the safety of one of these agents, and the danger of others is announced, to say nothing of the occasional discovery and alleged great advantage of new agents of this class. Of the latter it may be said, in passing, that their only claim entitled to respect and belief on the part of the profession thus far, is based upon the fact that they are novel. The action and adaptation to particular cases of anæsthetics other than those which head this article are comparatively obscure, and except in the hands of their discoverers, have little place in the armamentarium of surgeons.

It is curious to note the oscillations which the first of these, ether, has undergone in the opinion of operators. In this country it has been from the first the favorite agent for producing insensibility during the performance of what would be otherwise excessively painful, and in some instances, impossible operations. But more recently there has been a movement among American surgeons to replace it, in some cases, by chloroform, following the English and German line of thought and practice in this particular. On the other hand, some of our European confreres, attracted by the quite noticeable absence of reported accidents occuring during either anæsthesia, and attributed to the latter, have turned their attention to the uses and advantages of this anæsthetic agent.

In the Geneva clinic, Julliard seems to have led the way among European surgeons in the abandonment of chloroform, and the substitution therefor of ether. His results were of so highly satisfactory a character as to arrest the attention of Dumont.* This surgeon has now used sulphuric ether for over two years, to the exclusion of chloroform, and his experiences are entirely corroborative of those of Julliard. He expresses himself as being entirely in favor of this agent, and that, too, in very decided terms, claiming for it all that American surgeons have claimed for it in regard to its superiority over chloroform. With increasing use and familiarity with this means of producing surgical narcosis, Julliard and Dumont became quite as expert in its use as their colleagues upon this side of the Atlantic, frequently succeeding in producing complete surgical narcosis in from one to two minutes. The freedom from dangerous and threatening symptoms occurring in the course of the administration, in the hands of Dumont, is also a striking feature of this surgeon's experi-

* Korrespondenzblatt f. Schweizer Aertzte, 1889. No. 23.

ence. For instance, he reports that in 182 administrations in but a single instance was there any necessity for anxiety regarding the patient's condition. In this patient, an old subject suffering from chronic bronchitis, collapse occurred. The conclusions of Dumont in regard to the propriety of administering sulphuric ether to persons suffering from pulmonary affections are entirely in accord with those to which surgeons of extensive experience in the use of this agent have arrived, viz., that such must always be a source of more or less anxiety on the part of the administrator. In addition it may be noted, that it is also a rather universally acknowledged fact that the more acute the lesion of the air passages or pulmonary structure itself, the greater the danger.

There can be no gainsaying the fact that ether produces far greater irritation of the air passages than chloroform; and further, that the resistence to its administration, owing to the involuntary repugnance almost invariably shown to its vapor, on the part of patients, is somewhat difficult to overcome, and then stands in the way of an easy and comfortable transition from a state of consciousness to that of insensibility as compared with its rival. The assertion, however, I know, will be echoed by thousands who have used ether for many years, that these disadvantages are far more than counterbalanced by the decidedly greater safety of this agent.

It is a noticeable fact, yet one not easily accounted for, unless one may imagine a national determination to disparage anything which Germans have a decided leaning toward, that in those portions of Switzerland where the French element prevails, there is a growing interest in ether, and this agent has been steadily growing in favor as an anæsthetic in these districts.

Be that as it may, our German colleagues seem to have taken alarm at the increasingly large mortality attending chloroform administrations, and with their proverbially philosophical and inquiring turn of mind, have set out to settle the truth about this question, in their usual exact manner, by instituting a series of experiments upon the lower animals. It is now nearly two years ago since Ungar, discoursing upon this theme, claimed that animals, chloroformed for several hours at a time, and upon successive days, showed upon dissection indubitable evidences of fatty degenerations of, first, the heart and liver; second, of the kidneys and striped muscular structures; and third, of the gastric and mucuous membranes generally. These were attributed by Ungar to the direct poisonous effects of chloroform. More recently Strassman * has published some very interesting experiments bearing upon the question of the fatal after-effects of chloroform, in which he essentially corroborates the observations of Ungar, and sums up the results of his researches as follows:

1. After prolonged chloroformization in dogs, there can be demonstrated a fatty metamorphosis of the liver; the heart may partake of the same changes as a secondary result. Other organs are seldom affected. These changes consist of true fatty degeneration and not of fatty infiltration.

2. Subsequent to the usual chloroform narcosis, and when recovery thereform has apparently taken place, a fatal result is occasionally observed to occur.

3. Inasmuch as in the fatal cases the heart changes were found to be particularly well marked, these latter may reasonably be assumed to have been the cause of death.

4. In non fatal cases the evidences of degenerative changes are not found after several weeks.

5. These changes are particularly prone to occur in those in whom debilitating influences, such as hunger, loss of blood, etc., can reasonably account for the susceptibility to this undue action of the anæsthetic. In young and vigorous animals a greater power of resistance counteracts the tendency to these changes.

---

* Virchow's Archiv., Bd. 115.

These researches and published observations of both Ungar and Strassman, agreeing as they do in all essential particulars, point to but one conclusion. The charges brought against chloroform by its earlier opponents are more than sustained; for whereas it was supposed that the accidents during its administration were due to influences of a more or less functional character, it now transpires that, in addition to what has been looked upon either as a paralysis of the heart muscle through interrupted nerve force, or over-stimulation of the inhibitory apparatus, or both, we are to expect, in addition, true organic changes in the structure of the heart itself, as a result of the use of chloroform.

The deductions to be drawn from this presentation of facts would seem to be obvious, and yet, notwithstanding the apparently logical inferences which one would at the first glance draw, after careful study of the question, there are still some practical difficulties in the way of laying down any hard and fast rule regarding the choice as between chloroform and ether as an anæsthetic agent. It would seem, however, as if the following conclusions, as far as they go, might be justified:

1. As an anæsthetic for surgical operations, ether will be found to be less dangerous and more generally applicable.

2. If chloroform is to be used at all, it should be only for short operations or dressings otherwise painful. If necessity exists in any given case for the frequent exhibition of an anæsthetic, chloroform should give place to ether. Young and vigorous persons, and even those in advanced years who have not been subjected to debilitating influences, or are not the subjects of degenerative disease, may take chloroform with comparative safety; yet there is no reason why this same class may not take the safer anæsthetic, ether, as well.

3. In aged persons suffering from pulmonary affections, the administration may be commenced with chloroform; but, if it is found that the operation is to be a long one, ether should be substituted.

4. If degenerative disease of the kidneys and heart exist, and no pulmonary complication, ether should be used. If all of these conditions co-exist, judgment must be exercised as to which has a predominating influence, and the choice made accordingly. In any exent, the anæsthetic chosen under these circumstances should be used with great care.

I desire to refer to the pernicious habit of producing a mixed narcosis (chloroform and ether given simultaneously) simply to condemn it most unqualifiedly. Whatever good or evil resides in either agent should be kept apart, and the habit of uniting the one and at the same time attempting to prevent the undue influence of the other, must always remain a matter of uncertainty and doubt; the practice is, beyond doubt, unscientific, and should always be discouraged to the last degree.

The habit of administering atropia hypodermically, prior to the administration of the anæsthetic has received the sanction of authority and I know of no valid objection to urge against it. In ether narcosis it certainly lessens the tendency to profuse secretions from the mucous membranes of the air passages, and Bartholow's assertion that it acts as a stimulant to the respiratory centers seems to be borne out by experience. There are some advantages in combining morphia with this agent, particularly where it is deemed best to use chloroform as the anæsthetic agent. Without doubt it seems to lessen the amount of chloroform necessary to produce and maintain anæsthesia, and correspondingly lessens the danger to be apprehended from the use of the latter.

There have been some attempts made during the past four years to popularize in the profession the use of nitrous oxide as an anæsthetic for general surgical use. The name of Paul Bert, of France, is prominently associated with these attempts, but his published observations tended to discourage, rather than encourage, surgeons in the use of this agent. The reason for this resides in the fact that Bert sought to prove that for the purpose of its proper and safe administration for a protracted time, it is necessary that a specially prepared chamber be used, and that a fixed and certain atmospheric pressure be maintained during the administration. With ether and chloroform always at command, no surgeons would submit to such elaborate preparations, involving as they do an outlay of money, time and energy not warranted by the advantages to be obtained by the use of the gas. I have always entertained some doubt as to the necessity, in the use of this agent, for such extraordinary precautions, and I therefore hailed with delight the assertion of Dr. Geo. W. Brush, of Brooklyn, who has had many years' experience in the use of nitrous oxide, administered for dental operations, that it could in most cases be utilized for prolonged operations, with the patient in the recumbent position. The wide-spread belief in the innocuousness of the gas, the utter absence of depression arising from its use, the absence of any irritating qualities, as well as taste and smell, together with the ease of its administration and rapidity with which patients recover from its anæsthetic effects have always been attractive qualities to me; but I confess that the appearances of ghastly cyanosis and impending suffocation which the patient presents while under its influence, and which to my mind could only be explained by an accumulation of carbonic dioxide in the blood, deterred me from giving the agent that fair and unprejudiced trial which it would seem to deserve.

Yielding, however, to the assurances of Dr. Brush that, whatever the cyanosis might be due to, it certainly was not a sign of impending dissolution, I consented to allow of its trial in a very important operation, the pioneer of its kind, that of modified laryngectomy after Cohen's proposition. Dr. T. R. French, our esteemed laryngologist has seen the case, one of typical epithelioma of the left ventricular band and vocal cord, with me, and it was upon his suggestion that I entertained Dr. Brush's proposition, with whose experience in nitrous oxide administration I was not at that time familiar, to allow him to demonstrate the feasibility, (even under the adverse circumstances of being compelled to administer the gas through a tracheotomy tube, and keeping the nostrils plugged and mouth covered), of keeping the patient in a condition of safe anæsthesia for a length of time sufficient for the purposes of this operation. The result was a perfect revelation to me. The operation, together with the elaborate packing of the wound, securing of the stump of the trachea and fixation of a feeding tube in the opened œsophagus, occupied just one hour and forty minutes; during the whole of this time the anæsthesia was maintained, and at its close the patient, whose pulse had never wavered, but on the contrary, preserved its force and rate the same as before she came upon the table, became fully conscious within two minutes after the administration of the gas ceased. Had she not been prevented from doing so, she would have insisted upon her ability to walk from the operating-room to her own bed. No elaborate preparation was needed and the only departure from the apparatus ordinarily employed by the dentists, consisted of a screw coupling by means of which the tubing which conveyed the gas could be attached to the tracheotomy tube.

But the cyanosis, the ghastly appearance, even worse than that presented when the grim monster himself has taken possession of the patient, was present all the time; and I am not ashamed to say that, in the absence of any rational explanation of the condition, it was all that I could do to preserve my equanimity during this trying period. In spite of Dr. Brush's assurances, I felt far more comfortable, and whatever credit my colleagues who witnessed that operation may be inclined to give me for its successful completion, they will never know how much I was handicapped by that ashen gray, death-like upturned face within a few inches of my own during the whole time.

But why this peculiar condition, that which nothing can be more horrible? If it was not the result of the accumulation of carbonic dioxide in the blood, what was it? It was small comfort to know that one's patient was not being suffocated with one agent, while the suggestion was constantly before one's eyes that either that or something else was threatening to suspend his animation or destroy his life. Dr. Brush's assertion that they always look badly under its influence is somewhat reassuring, but his suggestion that the appearances were due to some chemical changes in the blood itself, only leads one to dread the possibility of there being some ultimate, if not immediate, danger resulting from its administration. Chemical combinations which could produce such changes in the circulating medium, even temporarily, are not to be disregarded.

With this case yet fresh in my mind, I sought some explanation for this somewhat anomalous state of apparently threatened death, and yet assured safety. In the course of my inquiries I found some observations by Ulbrich referred to. This observer, I found, had used the spectroscope for the purpose of determining the relation which nitrous oxide bore to the blood during its administration. As a result of his researches, Ulbrich declared that there was a chemical union between nitrous oxide and the hæmoglobin. These observations, unless it could be shown that this union was not of a character to destroy or even abrogate temporarily the funation of the hæmoglobin, would still leave the question an unsettled one, provided they were confirmed.

Spectroscopic analysis of the blood were not new, for in 1871, Preyer, of Jena, made similar researches. Buxton* directed his spectroscopic researches particularly toward the physiological effects of nitrous oxide. Later, McMunn†

* Korrespondernblatt f. Zahnarzte, Berlin, 1887.
† Internat. klin. Rundschau, Vienna, 1884, No. 4.

made spectroscopic experiments upon the blood. But the most carefully conducted experiments upon the relation which nitrous oxide bore to the blood seem to have been those of Rothmann, ‡ and these may be looked upon as being the most reliable studies upon the subject up to the present time. §

In the first place, Rothmann, with true german instinct, set out to criticise the methods of the only one of these observers who drew any definite conclusions from his studies, namely, Ulbrich. He declares the experiments of the latter to be utterly unreliable, for the reason that he made use of concentrated solutions of the blood elements. In following Ulbrich's methods, it was found that as a result of this concentration the spectroscopic absorption bands are shown broader, darker, or quite unrecogn izable. Rothmann's first step was to establish beyond a doubt the exact spectrum of the hæmoglobin when in the full performance of its function, *i. e.*, combined with oxygen. He then established the fact that the spectrum of blood, loaded or saturated with nitrous oxide, possessed precisely the same spectrum as oxyhæmoglobinic blood. This is a point of incalculable importance, for inasmuch as the hæmoglobin is enabled to continue its function, in spite of the presence of the gas in the circulating medium, no harm can come from the continued administration of the latter during long operations. Upon suspending the inhalation of the gas, the processes of oxydation are at once resumed as the atmospheric air is breathed.

The favorable experiences of the dental branch of the profession extending over many years in the administration of nitrous oxide for the performance of short operations will always warrant its use for such purposes. The results of Rothmann's operations, which, if they do not explain the condition of apparent cyanosis, at least demonstrate

‡ Vierteljahrschrift f. Zahnheilkunde, 1888, Hft. 3.
§ August, 1889.

that the essential blood elements, far from being in the condition which characterizes a suspension of their function (carbo-hæmoglobinic blood), are in precisely the opposite state (oxo-hæmoglobinic blood), and that, hence, the administration of the gas may be kept up for a length of time sufficient for almost any operation with perfect safety.

I should not fail, in this connection, to call attention to what may be a source of inconvenience in the use of this agent in general surgical operations. I refer to the altered color of the blood as it flows from the cut vessels, and the difficulty in some localities of distinguishing by its color arterial flow from simple venous or capillary oozing. An arterial twig may, for several reasons, not always give off a *per saltum* stream, and yet furnish sufficient blood to embarrass the operation and require the clamp, and perhaps a ligature as well. While slight pressure will suffice for simple capillary oozing, it is a waste of time to resort to this in the case of a flow from an arterial branch. If these two are combined (arterial flow and capillary oozing) and the location of the artery is not marked by its usual interrupted stream, one can readily see how the operation may be delayed pending an effort to identify the latter.

Another point occurs to me. In the cases in which the gas was administered for me, not all gave me such a favorable experience as the one above related. In at least one, a case in which I designed to remove a portion of the lower jaw for necrosis, it was found impossible to bring the patient under its influence. Ether was then used, but, whether from the previous efforts with the nitrous oxide or from some peculiar idiosyncrasy on the part of the patient, it was found impossible to control her powerful efforts at resistance without exerting undue force. I was finally compelled to order the substitution of chloroform. Under this agent the operation was brought to a satisfactory conclusion. This inability to anæsthetize the patient is a rare one, according to dental operators.

Finally, a peculiar condition of muscular rigidity, which is not abolished by pushing the anæsthetic, as in the case of other agents, but which rather increases as the narcosis deepens until it finally extends to the muscles of respiration, is sometimes noticed. When this latter circumstance is observed, it is an imperative demand for a suspension of the anæsthetic. This very seldom troubles our dental friends, for it is just at this stage that, by a dexterous twist, they accomplish the purpose for which the anæsthetic was administered. But in an operation which is to be a prolonged one, even if the rigidity does not extend to the respiratory muscles, it may become a source of serious embarrassment to the operator, particularly under circumstances where a relaxed condition is absolutely necessary, such as operations about the anus, rectum, perinæum, etc. This complication has likewise occurred to me.

Neither clinical experience nor experimental observation has yet demonstrated to the entire satisfaction of surgeons the exact conditions under which each of these anæsthetics may be most advantageously employed. My advice is to attempt no operation requiring anæsthesia with but one of these agents only at hand; and if it be decided beforehand to use nitrous oxide, it would be but a wise precaution to have both of the others within reach.—*Brooklyn Medical Journal.*

